# MPA Modulates Tight Junctions' Permeability via Midkine/PI3K Pathway in Caco-2 Cells: A Possible Mechanism of Leak-Flux Diarrhea in Organ Transplanted Patients

**DOI:** 10.3389/fphys.2017.00438

**Published:** 2017-06-26

**Authors:** Niamat Khan, Lutz Binder, D. V. Krishna Pantakani, Abdul R. Asif

**Affiliations:** ^1^Institute for Clinical Chemistry/UMG-Laboratories, University Medical CenterGoettingen, Germany; ^2^Department of Biotechnology and Genetic Engineering, Kohat University of Science and TechnologyKohat, Pakistan; ^3^German Center for Cardiovascular Research, Partner Site GoettingenGoettingen, Germany

**Keywords:** epigenetics, promoter assay, MPA, PI3K, midkine, AKT, inhibition

## Abstract

Mycophenolic acid (MPA) is prescribed to prevent allograft rejection in organ transplanted patients. However, its use is sporadically linked to leak flux diarrhea and other gastrointestinal (GI) disturbances in around 75% of patients through yet unknown mechanisms. Recently, we identified Midkine as a modulator of tight junctions (TJs) permeability in MPA treated Caco-2 monolayer. In the present study, we investigated the possible involvement of Midkine dependent PI3K pathway in alteration of TJs under MPA treatment. Caco-2 cells were grown as monolayer to develop TJs and were treated for 72 h with DMSO (control) or MPA in presence and absence of Midkine inhibitor (iMDK) or PI3K inhibitors (LY/AMG). Caco-2 monolayer integrity was assessed by transepithelial electrical resistance (TEER) and FITC-dextran assays. Our functional assays showed that PI3K inhibitors (LY/AMG) can significantly inhibit the compromised TJs integrity of MPA-treated Caco-2 cells monolayer. Chromatin immunoprecipitation analyses showed a significant epigenetic activation of M*idkine, PI3K, Cdx-2*, and *Cldn-2* genes and epigenetic repression of *Cldn-1* gene after MPA treatment. The MPA-induced epigenetic alterations were further confirmed by mRNA and protein expression analysis. Collectively, our data shows that PI3K pathway as the downstream target of Midkine which in turn modulates p38MAPK and pAKT signaling to alter TJs permeability in Caco-2 cell monolayers treated with MPA. These results highlight the possible use of either Midkine or PI3K inhibitors as therapeutic agents to prevent MPA induced GI disturbances.

## Introduction

Immunosuppressive drugs (ISDs) are prescribed to lower the body's ability to reject transplanted organs (Gummert et al., 1999). Mycophenolic acid (MPA) is an ISD available as mycophenolate mofetil (MMF), and enteric-coated mycophenolate sodium (EC-MPS), which selectively decreases the *de novo* synthesis of guanine nucleotide pool by inhibiting the enzymatic activity of inosine-5′-monophosphate dehydrogenase-2 (IMPDH-2; Sintchak and Nimmesgern, 2000) and halt the proliferation of lymphocytes at S-phase (Weigel et al., 1999).

Clinical data show the occurrence of a significant number of drug-induced diarrhea incidences in liver and kidney organ transplanted patients receiving MPA therapy (Helderman and Goral, 2002; Malinowski et al., 2011; Krones and Hogenauer, 2012). Diarrhea can result in dehydration and discomfort in transplanted patients. Although dose reduction may decrease the chance of diarrhea, it may also increase the rate of acute graft rejection. To overcome the diarrhea issue, two possibilities were proposed, in which either to quantitatively assess and compare the overall diarrheogenic potential or to explore the cellular mechanism(s) of diarrhea of MPA (Pescovitz and Navarro, 2001). The first possibility is very difficult or may even be impossible because of specific toxic effects of any ISD cannot be dissociated from the potential contribution of other factors such as drug–drug interactions (Pescovitz and Navarro, 2001). On the other hand, understanding cellular mechanism(s) could help to describe the pathophysiology of ISD-induced diarrhea and to explore potential anti-diarrheal intervention.

Tight junctions (TJs) are complex structures at the apical region of adjacent cells of epithelial monolayer that lines gastrointestinal (GI) tract (Tsukita et al., 2001). TJs control paracellular movement of molecules and ions across the monolayer. Different types of physiological and pathophysiological stimuli deregulate several pathways such as PKC (Fasano et al., 1995; Seth et al., 2008), PI3K (Suzuki et al., 2011), MLCK (Marchiando et al., 2010), Rho/ROCK (Le et al., 2014), and p38MAPK (Seth et al., 2008; Al-Sadi et al., 2013), which are involved in TJs regulation. Altered regulation of these pathways can lead to the alteration in TJs proteins expression and/or distribution resulting in altered TJ assembly and/or increased permeability (Catalioto et al., 2011) and consequently causes diarrhea (Hodges and Gill, 2010). Previously, we have reported that the inhibition of p38MAPK pathway in MPA treated Caco-2 cells results in partial prevention of increased TJ permeability. Subsequently, we identified an increased expression of Midkine protein in MPA-treated Caco-2 cells and that the inhibition of Midkine could completely prevent MPA-mediated TJ permeability (Khan et al., 2016). Midkine is a growth factor implicated in the etiology of inflammatory diseases (Muramatsu and Kadomatsu, 2014). In HepG2 cells, Midkine was found to exclusively localize to the nucleus as well as to the nucleolus (Dai et al., 2005) and involved in the transcription of 45 rRNA gene (Dai et al., 2008). In disease model study, Midkine was observed to drive lung cancer through activation of PI3K pathway (Hao et al., 2013). Midkine also promotes growth, proliferation, and self-renewal of embryonic stem cells (ESCs) via PI3K pathway that show correlation between ESCs and cancer (Yao et al., 2010). Interleukin-6 (IL-6) is known to increase TJ permeability via PI3K pathway in inflammatory bowel diseases (Suzuki et al., 2011). Based on above evidences, we hypothesized that MPA may alter the TJ assembly leading to increased permeability of epithelial cells via Midkine mediated PI3K pathway, which may cause diarrhea in organ transplanted patients.

## Materials and methods

### Cell culture

Human colon adenocarcinoma (Caco-2), an intestinal epithelial cell line, cells were purchased from DSMZ (German collection of microorganisms and cell culture, Braunschweig, Germany) and grown in culture flasks at 37°C with 5% CO_2_ and 95% humidity in DMEM medium supplemented with 10% FBS, 1% Penicillin/Streptomycin, and 1% non-essential amino acids. All experiments were performed with Caco-2 cells which were in between 15 and 25 passages. Caco-2 cells form differentiated and polarized confluent monolayer. This cell line has been regularly used to study the barrier function, drugs transportation across the monolayer, and pathophysiology of epithelium (Sambuy et al., 2005). Confluent monolayer were obtained within 3–5 days (d) when cultured with cell seeding density (2 ^*^ 10e5 cells/cm^2^). Post-confluent Caco-2 monolayers were further grown for 13 d prior to treatment and medium was changed every other day. In our previous studies, we found that therapeutic concentration of MPA alter TJ assembly in Caco-2 cell monolayer, but did not activate apoptotic pathway to compromise cell viability (Qasim et al., 2014). Therefore, we applied therapeutic concentration of MPA in all experiments performed in this study.

### Inhibitory assays

Stock solutions of Midkine inhibitor (iMDK, The Netherlands), PI3K inhibitors (AMG-511, ChemieTek, USA and LY294002, InvivoGen, USA), and p38MAPK inhibitor (SB203580, InvivoGen, USA) were prepared in DMSO. Inhibitor experiments were performed using working concentration of iMDK (25 nM), PI3K inhibitor AMG-511 (5 nM), PI3 inhibitor LY294002 (20 μM), and p38MAPK inhibitor SB203580 (10 μM) along with MPA (10 μM) therapeutic concentration.

### Experimental design

Six groups of differentiated and polarized Caco-2 cells monolayers were established and treated with MPA or iMDK+MPA or LY+MPA or AMG+MPA or SB+MPA or DMSO (control) for 72 h. Following the treatment, epigenetic, expression, and immuno-fluorescence experiments were performed according to established protocols (Qasim et al., 2014).

### Cell cytotoxicity assays

Cell viability assay of each group was performed using trypan blue dye exclusion assay as previously reported (Strober, 2001). Cell viability results were further confirmed by measuring cytotoxic marker Lactate Dehydrogenase (LDH) using cytotoxicity detection kit (LDH FS, Diaysys) and by following manufacturer's instructions. Results were presented as percentage and all values were normalized to control value of 100%.

### Caco-2 cells monolayer integrity

Following 72 h treatments, intactness of Caco-2 cells monolayer of each group was assessed through the most widely used TEER and FITC-dextran assays, as previously described (Khan et al., 2015).

### Primer design

Transcriptional Regulatory Element Database (TRED) was browsed to retrieve the promoter region sequences of the genes described in this study. Promoter based primers were designed by Primer3 (v. 0.4.0) and their specificity were confirmed by comparing with human genome in Human BLAT Search.

### Expression analysis

Total RNAs were isolated from Caco-2 cells of each group using Trizol method as previously described (Khan et al., 2016). For gene expression analysis, 1 μg RNA from each group was reversed transcribed into cDNA using cDNA kit (Invitrogen, USA) and used in real time PCR (Light Cycler® 480, Roche, Manheim, Germany) analysis.

The mRNA amount of the gene of interests in each sample was normalized to GAPDH, a housekeeping gene. Data was analyzed by comparative Ct method [2^−(ΔΔCT)^] and described as fold change between different groups (Schmittgen and Livak, 2008). Primer sequences are listed in Table S1.

### Western blot

Immunoblot analyses were performed as previously described (Khan et al., 2016). Briefly, whole cell lysate of each group was prepared using protein lysis buffer. Twenty micrograms of total proteins were separated by 12.5% SDS-PAGE and transferred onto PVDF membrane (Immobilon, Millipore, MA, USA) using Trans-Blot SD cell system (Bio-rad, Munich, Germany). After blocking with 5% milk, membranes were probed with primary antibody [2 μg/ml anti-Cldn-1 (mouse monoclonal (Invitrogen, Camarillo, CA, USA)]; 3 μg/ml anti-Cldn-2 [rabbit polyclonal (Novex, Life Technologies, Frderick, MD, USA)], AKT (Cell Signaling), and pAKT (p473AKT, Cell Signaling) in 5% BSA in TBS-T overnight at 4°C. Blotting for β-actin (Sigma, Mannheim, Germany) served as a loading control. Following overnight incubation with primary antibodies, membranes were washed and again incubated with appropriate HRP-conjugated secondary antibodies (Bio-Rad, Munich, Germany). Immunoreactive bands were visualized with enhanced chemiluminescence (GE, Buckinghamshire, UK) according to the manufacturer's recommendation. The densities of the specified protein bands between different groups were quantified using ImageJ software, version 1.48/Java (NIH, USA).

### ChIP assay

ChIP assay was performed as previously described (Khan et al., 2015). Briefly, Caco-2 cells were fixed with 37% formaldehyde and untreated formaldehyde was quenched with glycine. An equal number of cells from each treatment group was lysed with cell lysis buffer (Red ChIP Kit™, Diagenode). Chromatin shearing was performed by Branson Sonifier 250 in shearing buffer according to the manufacturer's instructions (Red ChIP Kit™, Diagenode). After pre-clearing the sheared chromatin, chromatin of each treatment was immunoprecipitated with active histone mark antibody [H3K4me3, rabbit polyclonal (abcam, Cambridge, UK)] or repressive histone mark antibody [H3K27me3, (MerckMillipore, Billerica, MA, USA)] overnight at 4°C. In parallel, IgG (One Day Kit™, Diagenode) was used as an experimental negative control and input (1%) was used as a positive control. Antibody-chromatin complexes were washed with 1X ChIP washing buffer (One Day Kit™, Diagenode). DNA–protein cross-linking was reversed by degrading DNA binding proteins with proteinase K. DNA was purified with DNA slurry (One Day Kit™, Diagenode). ChIP-precipitated genomic DNA as a template was amplified by real time PCR (Roche, Manheim, Germany) in 20 μL SYBR green based reaction (For promoter based primers see details in Table S1). Real time PCR data was analyzed by input percent method as previously described (Khan et al., 2015).

### Immunofluorescence microscopy of TJs proteins

Caco-2 monolayers were established on Lab-Tek™ eight chamber slides (Nunc, Naperville, IL, USA) and treated for 72 with MPA or iMDK+MPA or DMSO. Monolayers were immunostained as previously reported (Qasim et al., 2014), but with minor modifications. Briefly, monolayers were carefully rinsed with PBS and fixed with 3.7% formaldehyde for 20 min at room temperature (RT). Monolayers were rinsed with PBS and Triton X-100 (0.2%) was used for permeabilization of the cells within the monolayer, then rinsed with PBS and blocked with 1% bovine serum albumin (BSA) for 30 min at RT. Each monolayer was incubated overnight with anti-Cldn-1 (3 μg/ml) and anti-Cldn-2 (2 μg/ml) at 4°C. After washing with PBS, each monolayer was incubated with secondary antibodies (anti-rabbit IgG conjugated with Alexa 488 and anti-mouse IgG conjugated with Cy3 dye (Molecular Probes, Eugene, OR, USA) in 1% BSA for 1 h at RT in dark. Fluorescence images were acquired using Axiovert 200M confocal microscope (Carl Zeiss, Jena, Germany).

### Statistics

Data are presented as means ± standard error of the mean (SEM) of at least three independent experiments, each containing duplicates or triplicates. Graphpad prism 5 (GraphPad, San Diego, CA) was used to analyse the data. The multiple comparisons were made using One-Way-ANOVA and Bonferroni post-tests. The value *P* ≤ 0.05 was considered statistically significant (^*^P < 0.05, ^**^P < 0.01, ^***^P < 0.001).

## Results

### MPA induces midkine-dependent epigenetic activation of PI3K, *Cdx-2*, and *Cldn-2* genes and repression of *Cldn-1* gene

**Figure 1:**
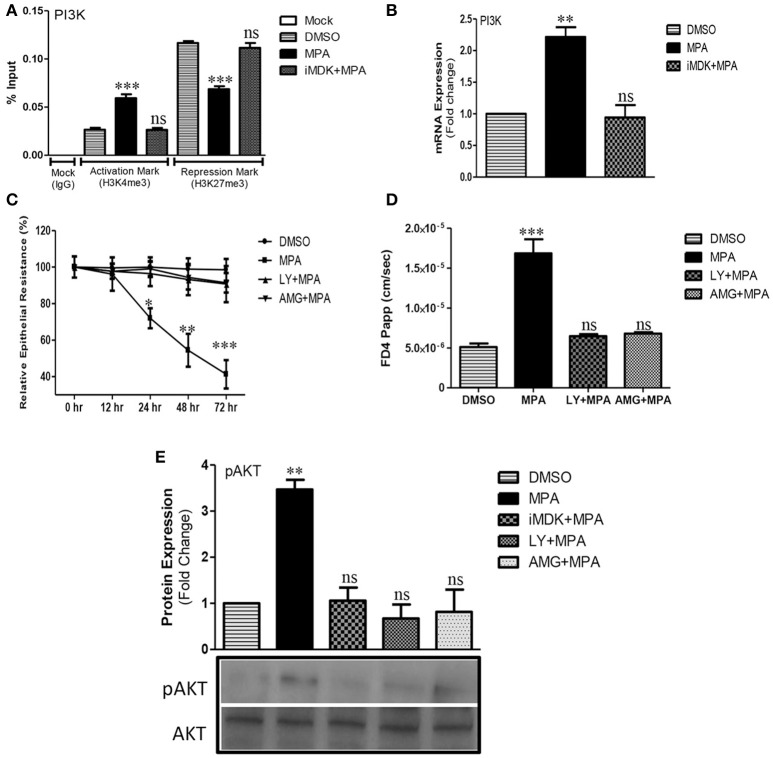
Influence of MPA on PI3K/AKT pathway and PI3K/AKT dependent modulation of TJs permeability. **(A)** ChIP-qPCR of gene activation mark (H3K4me3) and repression mark (H3K27me3) at the promoter region of PI3Kgamma gene in Caco-2 cells monolayer treated with either DMSO (Control) or MPA alone or in combination with Midkine inhibitor. **(B)** The mRNA expression of *PI3K* gene in Caco-2 cell monolayers treated with either DMSO (Control) or MPA alone or with Midkine inhibitor. *GAPDH* was used as a house keeping gene. **(C)** The influence of PI3K pathway inhibition on TJs permeability of Caco-2 monolayer treated with MPA. TEER was measured at 0, 12, 24, 48, and 72 h time points after DMSO (Control) or MPA with or without PI3K inhibitors (LY or AMG). **(D)** Paracellular FD4 dye flux assay results of Caco-2 monolayers treated with either MPA alone or in combination with PI3K inhibitors (LY or AMG). **(E)** Representative Western blot and densitrometric analysis of AKT/pAKT in Caco-2 cell monolayers treated with either MPA alone or in combination with Midkine inhibitor (iMDK), or PI3K inhibitors (LY or AMG). Data are shown as mean band density of pAKT relative to total AKT expression. **(A–D)** Statistically significant differences analyzed by ANOVA with Bonferroni post-test for multiple comparisons are indicated by ^*^P < 0.05, ^**^P < 0.01, ^***^P < 0.001. ns, non-significant. Error bars represent ± SEM (*n* = 3).

Different cellular signaling pathways (such as PI3K, PKCA, JNK, JunD, RhoA, etc.) are targeted by the infectious agents and cytokines to increase TJs permeability in several disease conditions and model systems (Amasheh et al., 2010; Al-Sadi et al., 2013; Le et al., 2014). We investigated the influence of MPA treatment on the regulation of these pathways through targeted epigenetic approach (promoter assays of the selected genes). Our epigenetic analyses showed that MPA treatment leads to epigenetic activation of PI3K (Figure 1A and Figure S1A). We analyzed the PI3K promoter epigenetic status by ChIP and identified significant enrichment of transcriptional activation mark (H3K4me3) and concomitant decrease in transcriptional repression mark (H3K27me3) after MPA treatment (Figure 1A). To confirm these results, we analyzed the PI3K mRNA expression and found it to be upregulated (Figure 1B). Following this, we performed Midkine-dependency analysis for PI3K epigenetic activation during MPA treatment and found that the inhibition of Midkine results in downregulation of PI3K expression and also the epigenetic silencing marked by increased H3K27me3 levels (Figures 1A,B). These results confirm that Midkine indeed regulates PI3K expression during MPA treatment.

To test the role of PI3K pathway in TJs regulation, we employed two PI3K inhibitors (LY or AMG). The LY is known to be effective at 20 μM in human epithelial cell lines (Caco-2, HT29, MCF10A cells, and T84 cells; Laprise et al., 2002; Porquet et al., 2011; Smyth et al., 2012; Elsum et al., 2013). AMG, was not previously tested in Caco-2 cells, hence we firstly established that 5 nM of AMG could be used without compromising the cell viability (Figures S2A,B). Then we assessed the role of PI3K pathway in TJ integrity using TEER and FD4 dye flux assays. Our results showed that MPA-mediated decreased TEER could be significantly blocked with pre-incubation of both PI3K pathway inhibitors (LY or AMG) in Caco-2 cells monolayers followed by co-incubation with LY+MPA or AMG+MPA (Figure 1C). Similarly, MPA-mediated FD4 dye flux was significantly blocked across the Caco-2 cell monolayer that was exposed to PI3K inhibitors (LY or AMG) 1 h prior to the co-incubation with therapeutic concentration of MPA (Figure 1D).

Protein kinase B (PKB) or AKT is a serine-threonine kinase that is activated by PI3K transducer and is involved in the regulation of different biological processes including angiogenesis, growth, metabolism, proliferation, and cell survival (Manning and Cantley, 2007). However, cytokines (such as TNF-α, IL-6) increase TJ permeability via PI3K/AKT pathway (Amasheh et al., 2010; Suzuki et al., 2011). Hence, we checked for the pAKT expression (phosphorylated AKT) by Western blot analysis and found that MPA treatment increases the pAKT levels, which can be counteracted by pre-treatment with either Midkine or PI3K inhibitors (Figure 1E).

### MPA activates Cdx-2 expression via Midkine/PI3K pathway

**Figure 2:**
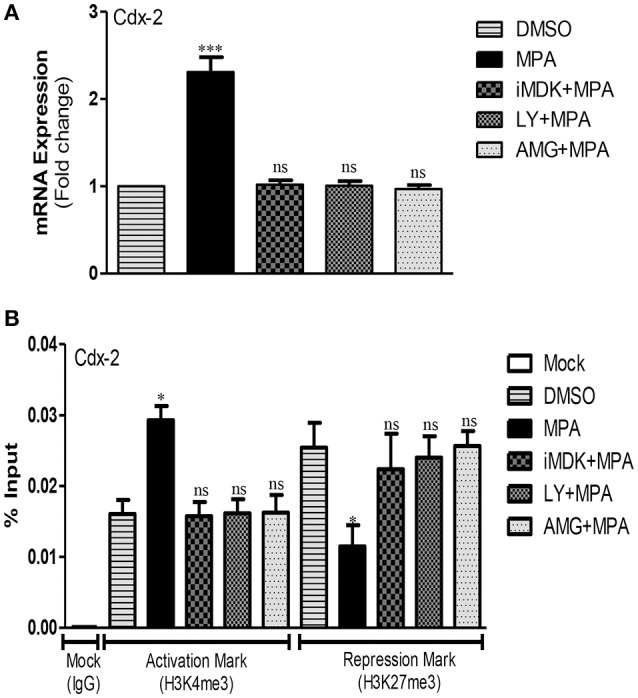
Activation of Cdx-2 gene via Midkine/PI3K pathway after MPA treatment. **(A)** qRT-PCR results of *Cdx-2* mRNA expression in control (DMSO) and different treatment groups. *GAPDH* was used as a house keeping gene. **(B)** The ChIP-qPCR of activation mark (H3K4me3) and repression mark (H3K27me3) at the promoter region of *Cdx-2* in Caco-2 cells monolayer treated with either MPA alone or in different treatment groups. DMSO treated cells were used as a control. Statistically significant differences analyzed by ANOVA with Bonferroni post-test for multiple comparisons, are indicated by ^*^P < 0.05, ^***^P < 0.001. ns, non-significant. Error bars represent ± SEM (*n* = 3).

Cdx-2 is a transcription factor involved in the regulation of early differentiation and maintenance of intestinal epithelial cells (Bhat et al., 2012). Increased production of IL-6, a cytokine known to increase TJ permeability in IBD (Suzuki et al., 2011), is reported to enhance the expression of Cdx-2 via PI3K pathway that in turn transcriptionally activates Cldn-2 and consequently increases TJ permeability for cation molecules (Suzuki et al., 2011). Whether MPA modulates *Cdx-2* expression via PI3K pathway in MPA treated Caco-2 cells is not known. To address this question, we performed qRT-PCR analysis and found that *Cdx-2* mRNA levels increase significantly in Caco-2 cells treated with MPA (Figure 2A). Interestingly, the increased *Cdx-2* expression is prevented by inhibiting either Midkine or PI3K signaling pathways (Figure 2A). Then, we performed epigenetic analysis of *Cdx-2* promoter region and found that MPA treatment significantly increases H3K4me3 mark and concomitantly decreases H3K27me3 (Figure 2B), indicating the activation of *Cdx-2* gene. In line with the gene expression data, iMDK or PI3K inhibitors (LY or AMG) significantly prevented the increased promoter activation of Cdx-2 gene in MPA-treated cells as compared to control (Figure 2B). These results indicate that *Cdx-2* is epigenetically activated after MPA-treatment via Midkine/PI3K pathway.

### MPA-mediated altered expression of Cldn-1 and Cldn-2 are prevented by Midkine or PI3K inhibitors

**Figure 3:**
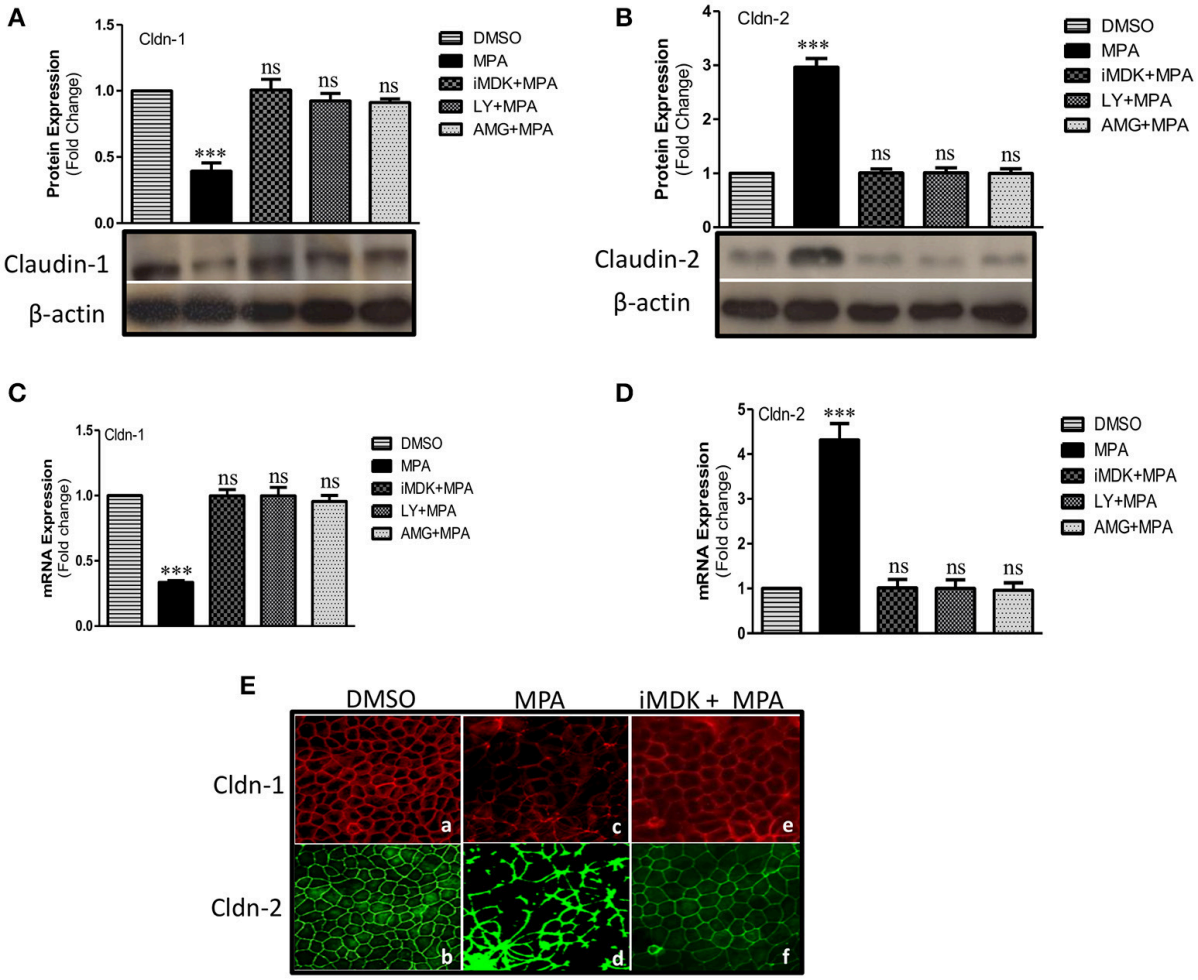
Influence of Midkine mediated PI3K pathway on TJ structural proteins (Cldn-1, -2) in MPA treated Caco-2 cells. **(A,B)** Western blot analysis of TJ proteins (Cldn-1, -2) in Caco-2 cell monolayers treated with MPA alone or in combination with Midkine/PI3K inhibitors (iMDK/LY/AMG). Immunoblots were probed with antibodies against TJ proteins **(A)** Cldn-1 and **(B)** Cldn-2. β-actin was used as a loading control. **(C,D)** qRT-PCR analysis of *Cldn-1* and *-2* gene expression in Caco-2 cell monolayers treated with MPA or Midkine/PI3K inhibitors (iMDK/LY/AMG). GAPDH was used as a house keeping gene and differences between groups were analyzed by ANOVA with Bonferroni post-test. The values were expressed as means ± SEM (*n* = 3). ^***^P < 0.001, or ns, non-significant when compared with control cells. **(E)** Differentiated/polarized monolayer of Caco-2 cells were treated with either DMSO, MPA alone or in combination with Midkine inhibitor (iMDK) for 72 h, fixed, permeated, and stained for Cldn-1 and Cldn-2. The distribution of Cldn-1 and Cldn-2 in differentiated and polarized Caco-2 monolayer exposed to DMSO (a,b), or MPA (c,d) or iMDK+MPA (e,f) is shown.

Cldn-1 and Cldn-2 are important components of TJs assembly. Cldn-1 is also known as pore-sealing Claudin therefore its expression is in inverse relation with the TJs permeability (Will et al., 2008; Khan and Asif, 2015). On the other hand, Cldn-2 is known as pore-forming Claudin and its expression is directly related to TJs permeability (Suzuki et al., 2011; Khan and Asif, 2015). Previous reports show that PI3K dependent down regulation of Cldn-1 and upregulation of Cldn-2 that in turn increase TJ permeability in different disease models (Kojima et al., 2008; Wang et al., 2011; Nomura et al., 2014). Our expression analysis showed that MPA-treatment significantly decreases the expression of pore-sealing Cldn-1 protein, while it increases the expression of Cldn-2 (Figures 3A,B). However, MPA mediated altered Cldn-1 and Cldn-2 expression was significantly blocked in Caco-2 cells monolayer pre-treated with iMDK or with LY or AMG (Figures 3A,B). In agreement with the protein expression data, qRT-PCR analysis further confirmed that MPA decreases Cldn-1 mRNA expression while it increases Cldn-2 mRNA expression (Figures 3C,D). Moreover, the altered Claudin's expressions could be prevented by pre-treatment of Caco-2 cell monolayers with either Midkine or PI3K inhibitors (Figure 3C). Activation of Cldn-2 and inactivation of Cldn-1 were further confirmed at epigenetic level (Figure S3).

Next, we performed immunostainings to examine the junctional distribution of Cldn-1 and Cldn-2 in Caco-2 cell monolayer. We observed a uniform and continuous pattern of Cldn-1 and Cldn-2 staining at the cell junctions in control cells (Figure 3E). Intriguingly, MPA-treatment not only resulted in decreased expression of Cldn-1, but also disrupted its distribution (Figure 3E). On the other hand, MPA treatment increased the expression of Cldn-2 as seen by marked increase in staining, however; the pattern of Cldn-2 is disrupted and is accumulated at some regions (Figure 3E). Caco-2 cell monolayers that were co-treated with iMDK along with MPA showed expression and distribution pattern that was quite similar to control cells (Figure 3E). Taken together, these results indicate that MPA treatment activates Midkine/PI3K pathway that in turn may alter the expression and distribution of TJs assembly proteins (Cldn-1 and Cldn-2) and consequently increase the permeability.

### p38MAPK pathway is activated by Midkine/PI3K signaling in MPA treated Caco-2 cells

**Figure 4:**
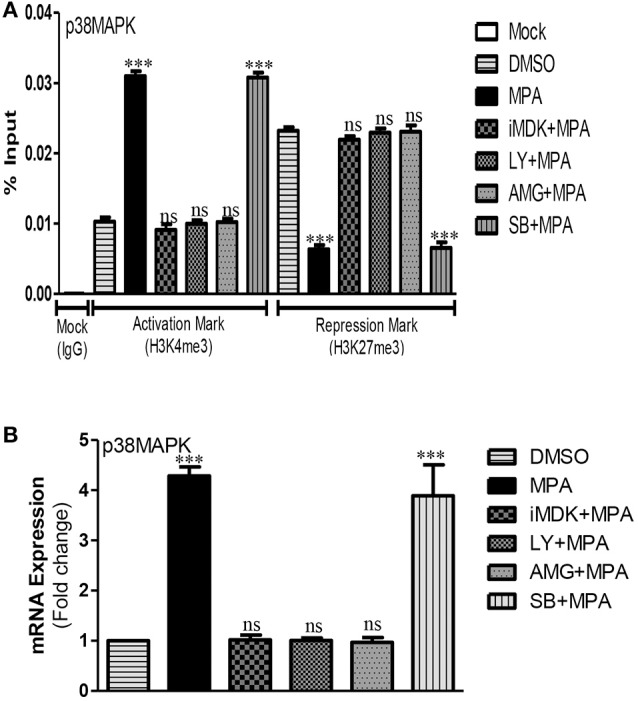
Midkine/PI3K dependent activation of p38MAPK pathway after MPA treatment. **(A)** The ChIP-qPCR data of activation mark (H3K4me3) and repression mark (H3K27me3) at the promoter region of p38MAPK in Caco-2 cell monolayers treated with DMSO (Control), MPA alone or in combination with Midkine, and PI3K inhibitors. **(B)** The mRNA expression levels of *p38MAPK* either in presence or absence of PI3K inhibitors in MPA treated Caco-2 cell monolayers as compared to control cells. Differences between groups were analyzed by ANOVA with Bonferroni post-test. The values were expressed as mean ± SEM (*n* = 3), whereas ^***^P < 0.001, ns, non-significant.

Previously, we reported that inhibition of p38MAPK pathway partially prevented MPA mediated increase of TJ permeability in Caco-2 cells monolayer (Khan et al., 2015). In the current study, we investigated cross talk between PI3K and p38MAPK pathways after MPA-treatment. Our results showed that MPA significantly activates *p38MAPK* gene at epigenetic level (Khan et al., 2015). MPA-mediated epigenetic activation of p38MAPK was significantly blocked in cells that were pre-incubated with Midkine or PI3K inhibitors (Figure 4A). Similar to this, the mRNA expression analysis data confirmed that inhibition of Midkine or PI3K is sufficient to counteract MPA-mediated changes and to restore normal levels of p38MAPK (Figure 4B).

## Discussion

Intestinal epithelium is gated by intercellular junctions such as gap junctions, adherens junctions, desmosome junctions, and TJs (Lisewski et al., 2008; Khan and Asif, 2015). TJs control paracellular pathway between the adjacent cells (Chen et al., 2006) and are especially important in preventing translocation of infectious agents and toxins from the lumen into the blood stream (Groschwitz and Hogan, 2009). Defects in TJs assembly are key factors associated with IBD and other gut inflammatory conditions (Lei et al., 2014). In the present study, we have characterized the disease-relevant role of Midkine dependent PI3K pathway that consequently induces TJs permeability in MPA treated Caco-2 cells monolayer. Midkine, a heparin-binding growth factor, plays an important role in the pathogenesis of inflammatory and malignant diseases (Muramatsu, 2014). The interesting aspect of this study is the Midkine dependent activation of *PI3K, Cdx-2, Cldn-2, p38MAPK* genes and inactivation of *Cldn-1* gene at the epigenetic, mRNA, and protein level in MPA treated Caco-2 cells. Furthermore, we analyzed the effects of Midkine or PI3K inhibition in MPA-treated confluent monolayer of Caco-2 cells and observed a protective role of Midkine and PI3K inhibitors (iMDK or LY or AMG) in MPA-treated cells.

Recently, we reported that inhibition of Midkine significantly blocks MPA-mediated increased TJs permeability in Caco-2 cells (Khan et al., 2016). Midkine is known as the upstream regulator of PI3K pathway (Hao et al., 2013). PI3K is a family of enzymes involved in different cellular functions including cell differentiation, growth, proliferation, motility, survival, and intracellular trafficking (Hawkins and Stephens, 2015). PI3K is also known to regulate epithelial barrier function via TJs modulation (Suzuki et al., 2011). We observed the enrichment of gene activation histone mark (H3K4me3) and simultaneously depletion of gene repressive histone mark (H3K27me3) at the promoter of *PI3K* gene in MPA treated Caco-2 cells.

Insulin, cytokines, growth, and survival factors are known to phosphorylate AKT through PI3K pathway (Burgering and Coffer, 1995; Franke et al., 1995; Suzuki et al., 2011). Phosphorylated AKT (pAKT) is able to regulate a large number of downstream targets including transcription factors (reviewed in Manning and Cantley 2007; Faes and Dormond, 2015). PI3K/AKT pathway is considered as a target in the therapy of IBDs such as Crohn's Disease (Tokuhira et al., 2015). IL-6 induces phosphorylation of AKT through PI3K pathway in Caco-2 cells and increases TJs permeability (Suzuki et al., 2011). To the best of our knowledge this is the first report that shows that Midkine up-regulation leads to phosphorylation of AKT via PI3K pathway and consequently alters TJs permeability in MPA-treated Caco-2 cells. Cdx-2 plays a critical role in the transcriptional regulation of intestinal genes (Boyd et al., 2010). Cdx-2 increases expression of pore-forming Cldn-2 in TJs assembly resulting elevated cations permeability via IL-6 dependent activation of PI3K/AKT pathway (Suzuki et al., 2011). We observed Midkine/PI3K dependent activation of Cdx-2 gene at promoter level and increased expression at mRNA level after MPA treatment.

Dynamic nature of TJs assembly partly depends upon the Claudin family members such as pore-forming and pore-sealing Claudins. Altered expression of Claudin family members significantly deregulates TJ permeability (Gunzel and Fromm, 2012). Overexpression of pore-sealing Claudin was reported to increase the TEER and to strengthen barrier integrity leading to decreased para cellular permeability in epithelial MDCK cells (Inai et al., 1999). While on the other hand, down regulation of pore-sealing Claudin such as Cldn-1 and overexpression of pore-forming Claudin such as Cldn-2 decrease TEER (Zhang et al., 2013). Decreased expression of Cldn-1 is associated with increased intestinal permeability in IBS patients (Bertiaux-Vandaele et al., 2011). Patients of IBDs such as ulcerative colitis and Crohn's disease have a mucosal barrier dysfunction that can be assessed by measuring the intestinal permeability (Gerova et al., 2011). Moreover, specimens of these patients showed higher expression of Cldn-2. Pro-inflammatory cytokines (TNF-alpha, IL-13, IL-6) are known to induce Cldn-2 expression in *in vitro* intestinal cell models as well as in *in vivo* mouse model (Heller et al., 2005; Weber et al., 2010; Suzuki et al., 2011). Such as IL-6 induces Cldn-2 expression via PI3K dependent pathway and increases TJ permeability (Suzuki et al., 2011). In agreement with earlier studies (Suzuki et al., 2011; Wang et al., 2011; Nomura et al., 2014), we show in this report for the first time that Midkine /PI3K pathway mediated down regulation of Cldn-1 and upregulation of Cldn-2 protein after MPA-treatment that leads to increase TJs permeability.

Decreased TEER-value and increased FD4 fluxes confirmed the modulatory role of MPA on barrier function. As TEER-value of monolayer inversely reflects the ion conductance through the TJs-controlled paracellular pathways, the alteration observed after MPA therapeutic concentration exposure highlights FD4 fluxes transportation across the Caco-2 monolayers. We observed significant decrease in TEER and increase in FD4 flux correlated with overexpression of Cldn-2 and downregulation of Cldn-1 in MPA treated Caco-2 cells. These results are consistent with the previous finding of *in vitro* and *in vivo* model studies such as MDCK cells show increase TEER with the overexpression of Cldn-1 that in turn decreases paracellular permeability (Inai et al., 1999; Amasheh et al., 2010). While Cldn-1 knockdown mice show loss of TJ barrier to water and macromolecules (Furuse et al., 2002).

TJs assembly proteins such as Claudins, Occludin are connected with F-actin based cytoskeleton through a scaffolding protein ZO-1 (Wittchen et al., 1999; Yokoyama et al., 2001; Muza-Moons et al., 2004). This anchoring property of ZO-1 protein is due to the PDZ motif in the N-terminal which interacts with PDZ-binding motif in the C-terminal domain of Claudins proteins (Morita et al., 1999; Fanning and Anderson, 2009). Recently, ZO-1 dependent interaction was observed between C-terminal tail of Cldn-1, Cldn-2, and Occludin proteins (Raleigh et al., 2011). ZO-1 knockdown cell studies show disruption of Claudins localization and barrier function (Ikenouchi et al., 2007; Fanning et al., 2012). Previously, our group has reported MPA-mediated modulation of F-actin based cytoskeleton via MLCK/MLC-2 pathway and redistribution of ZO-1 and Occludin from TJs assembly (Qasim et al., 2014). Recently we have reported a decreased expression of Occludin protein via p38MAPK dependent MLCK/MLC-2 pathway after MPA treatment (Khan et al., 2015). In this study, we observed irregular patterns of Cldn-1 and Cldn-2 in the TJs assemblies apart from their altered expression. This altered distribution pattern of Cldn-1 and Cldn-2 proteins in TJs assembly can be due to the disruption of F-actin based cytoskeleton, redistribution/downregulation of ZO-1 as well as redistribution/decrease expression of Occludin proteins in TJs assembly. Our results are consistent with previous findings such as (1) where *E. coli* heat stable toxin dissolves and condenses F-actin based cytoskeleton in T84 cells and alterations of F-actin based cytoskeleton were accompanied by redistribution of ZO-1, Cldn-1, and Occludin (Ngendahayo and Dubreuil, 2013). In an another *in vitro* study, alteration of F-actin based cytoskeleton and redistribution of Occludin, ZO-1, Cldn-1, and Cldn-2 were observed in the presence of RhoA, Rac1, and Cdc-42 enzymes (Bruewer et al., 2004). (2) ZO-1 knockdown and/or ZO-1 and ZO-2 double knockdown MDCK cells show disruption of the localization of Cldn-2 and Occludin at TJs (Van Itallie et al., 2009; Tokuda et al., 2014). (3) Cldn-1 and Cldn-2 recruit Occludin and reconstitutes TJs strands (Furuse et al., 1998). In the absence of Occludin, ZO-1 disappears from TJ and Cldn-1 is downregulated (Li and Mrsny, 2000).

In addition, *Salmonella* infection significantly increased the expression of Cldn-2 protein both in *in vitro* and *in vivo* model studies and modulated the localization of junctional Cldn2 protein (Zhang et al., 2013). Deprivation of glutamine not only decreases the expression of Cldn-1 protein but also altered distribution in TJs assembly via PI3K/AKT pathway in Caco-2 cells (Li et al., 2004; Li and Neu, 2009). IL-1β increases TJs permeability via p38MAPK/ATF-2 pathway in *in vitro* and *in vivo* model studies (Al-Sadi et al., 2013). Previously, we reported that MPA mediated increased TJs permeability can be partially prevented by blocking p38MAPK pathway (Khan et al., 2015). In this study, we observed Midkine/PI3K dependent activation of *p38MAPK* gene after MPA treatment. Consistent to the previous findings, the inhibition of Midkine/PI3K pathway completely blocked MPA modulated TJs permeability (Shahabuddin et al., 2006; Delgado-Vega et al., 2010).

Pro-inflammatory cytokines, infections, and chemical agents alter TJs-dependent permeability through different pathways, e.g., protein kinase C (PKC) and protein phosphatases (PP) regulate TJ assembly via altered phosphorylation of Occludin protein (Fasano et al., 1995). Expression of Thyroid Transcription Factor-1 (TTF-1) induces expression of Occludin and Cldn-1 in lung epithelial cell line (Runkle et al., 2012). *Salmonella* increases expression of Cldn-2 via JNK pathway while *Complyobactor jejuni* downregulates Cldn-4 in turn increases TJ permeability (Zhang et al., 2013). Depletion of polyamines disrupts TJs permeability via JunD dependent negative regulation of ZO-1 gene (Chen et al., 2008). Sodium butyrate and Naringenin enhances barrier functions through increased expression of Cldn-1 and Cldn-4, respectively, via transcription factor SP-1 (Wang et al., 2012; Noda et al., 2013). Loss of Hnf4A affects colonic ions transport via down regulation of Cldn-15 and causes IBD type chronic inflammation in mice (Darsigny et al., 2009). Decreased expression of Cldn-3 and Cldn-4 was observed in the colonic biopsies samples of IBD patients (Prasad et al., 2005). RhoA dependent increased TJs permeability is also reported by different studies (Terry et al., 2010). We did not find the activation of these pathways through our epigenetic analyses after MPA treatment which may be due to different methodology and differences in the used models.

**Figure 5:**
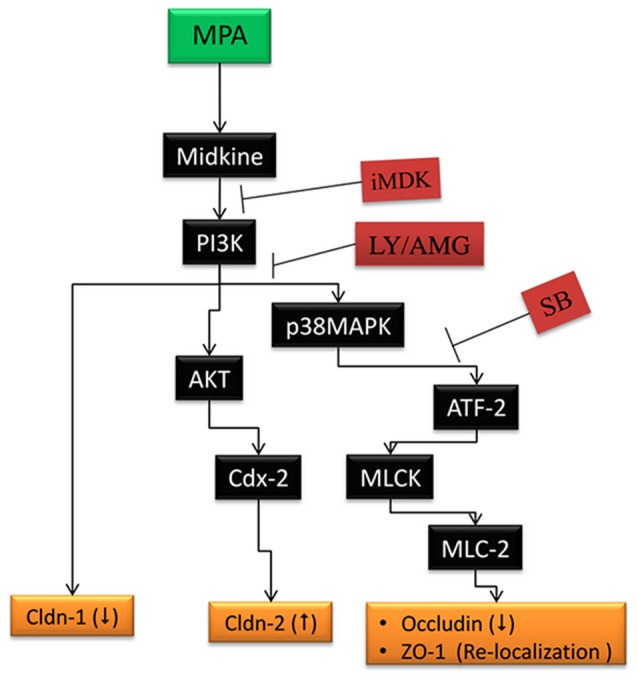
MPA-induced TJ assembly deregulation in Caco-2 cells monolayer. Our results indicates that Midkine activates PI3K/ p38MAPK dependent MLCK pathway and may reorganize F-actin based cytoskeleton leading to redistribution of ZO-1 and Occludin proteins as well as downregulation of Occludin protein. MPA, mycophenolic acid; p38MAPK, p38 mitogen activated protein kinase; ATF-2, activating transcription factor-2; MLCK, myosin light chain kinase; MLC-2, myosin light chain-2; TJ, tight junction; ZO-1, Zonula Occludins; PI3K, phosphatidylinositol-4,5-bisphosphate 3-kinase; Cldn-1, Claudin 1; Cldn-2, Claudin 2; iMDK, Midkine inhibitor; LY, LY294002; AMG, AMG-511; SB, 203580.

According to our knowledge, this is the first report which demonstrates the role of Midkine dependent TJ regulation via PI3K pathway in MPA treated Caco-2 cells. Based on our observations, we attempt to propose a model (Figure 5), in which, Midkine activates PI3K/p38MAPK dependent MLCK pathway and consequently reorganizes F-actin based cytoskeleton which leads to redistribution of ZO-1 and Occludin proteins as well as downregulation of Occludin protein. In parallel, Midkine protein increases the expression of Cldn-2 and decreases expression of Cldn-1 proteins in TJ assembly via PI3K pathway in MPA treated Caco-2 cell monolayer. Midkine and PI3K inhibitors (iMDK/LY/AMG) block the MPA-mediated altered TJ assembly in Caco-2 cells monolayers.

## Conclusions

This *in vitro* study demonstrates that exposure of confluent Caco-2 cell monolayers to the MPA therapeutic concentrations leads to Midkine dependent PI3K pathway activation that ultimately modulates the expression of Cldn-1 and -2 proteins and activates p38MPAK mediated MLCK/MLC-2 pathway and consequently increases monolayer permeability. Our results point to the potential of Midkine as a noninvasive permeability-based marker of leak flux diarrhea for the patient on MPA therapy after organ transplantation. The study provides first indications to the therapeutic ability of iMDK/LY/AMG which improved barrier function of Caco-2 cell monolayers under MPA stimulation. Additional studies are however necessary to understand the role of Midkine in MPA-treated cells and confirm these findings in relevant *in vitro* intestinal models.

## Author contributions

NK and AA conceived the study. NK and DP performed and analyzed the data. NK, DP, LB, and AA participated in interpretation and drafting the manuscript. DP and AA supervised the study. All authors read and approved the final manuscript.

### Conflict of interest statement

The authors declare that the research was conducted in the absence of any commercial or financial relationships that could be construed as a potential conflict of interest.

## Supplementary material

Figure S1 Regulatory effect of MPA treatment on the promoter of other genes involve in the regulation of TJ permeability. (**A,B)** Promoter activity of (*PKCA, JNK, RhoA, PP-2A, PP1, JunD, Sp-1, TTF-1, Hnf4A, Cldn-3, -4*, and *Cldn-15*) genes: ChIP assay was performed with antibodies specific to the activation mark (H3K4me3) or repression mark (H3K27me3) or IgG, followed by real time PCR analysis. Data was analyzed using % input to calculate the respective relative intensity of activation or repression mark in the MPA treated cells as compared to control (DMSO) cells. Differences between groups were analyzed by ANOVA with Bonferroni post-test. The values were expressed as means ± SEM (*n* = 3). Whereas, ns, non-significant.

Figure S2 Cytotoxic effects of PI3K inhibitor (AMG) in co-treatment with MPA on Caco-2 cells. **(A,B)** Caco-2 cells were grown for 13 days post-confluence and treated with MPA or AMG + MPA or DMSO for 72 h. Cellular viability was measured using trypan blue exclusion assay after 72 h of treatment. Results are expressed as percentage of DMSO control (100%). **(A)** Cell viability was further confirmed by measuring the LDH release in the culture media in percentage to the DMSO control (100%). **(B)** Differences between groups were analyzed by ANOVA with Bonferroni post-test. The values were expressed as means ± SEM (*n* = 3). Whereas, ^*^*P* < 0.05, ^**^*P* < 0.01, ^***^*P* < 0.001, and ns, non-significant.

Figure S3 Promoter activity of *Cldn-1* and *Cldn-2* genes. **(A,B)** ChIP assay was performed with antibodies specific to the activation mark (H3K4me3) or repression mark (H3K27me3) or IgG, followed by real time PCR analysis. Percentage input (% Input) method was applied to calculate the relative intensity of activation or repression mark. Differences between two groups were analyzed by one-way ANOVA with Bonferroni post-test. The values were expressed as means ± SEM. Whereas, ^**^*P* < 0.01, ^***^*P* < 0.001, and ns, non-significant.

Table S1 List of primers used in this study.
