# Supplementary figures and images for: MPA Modulates Tight Junctions' Permeability via Midkine/PI3K Pathway in Caco-2 Cells: A Possible Mechanism of Leak-Flux Diarrhea in Organ Transplanted Patients
Source: Front Physiol. 2017 Jun 26;8:438. doi: 10.3389/fphys.2017.00438 (PMC5483464; doi:10.3389/fphys.2017.00438)

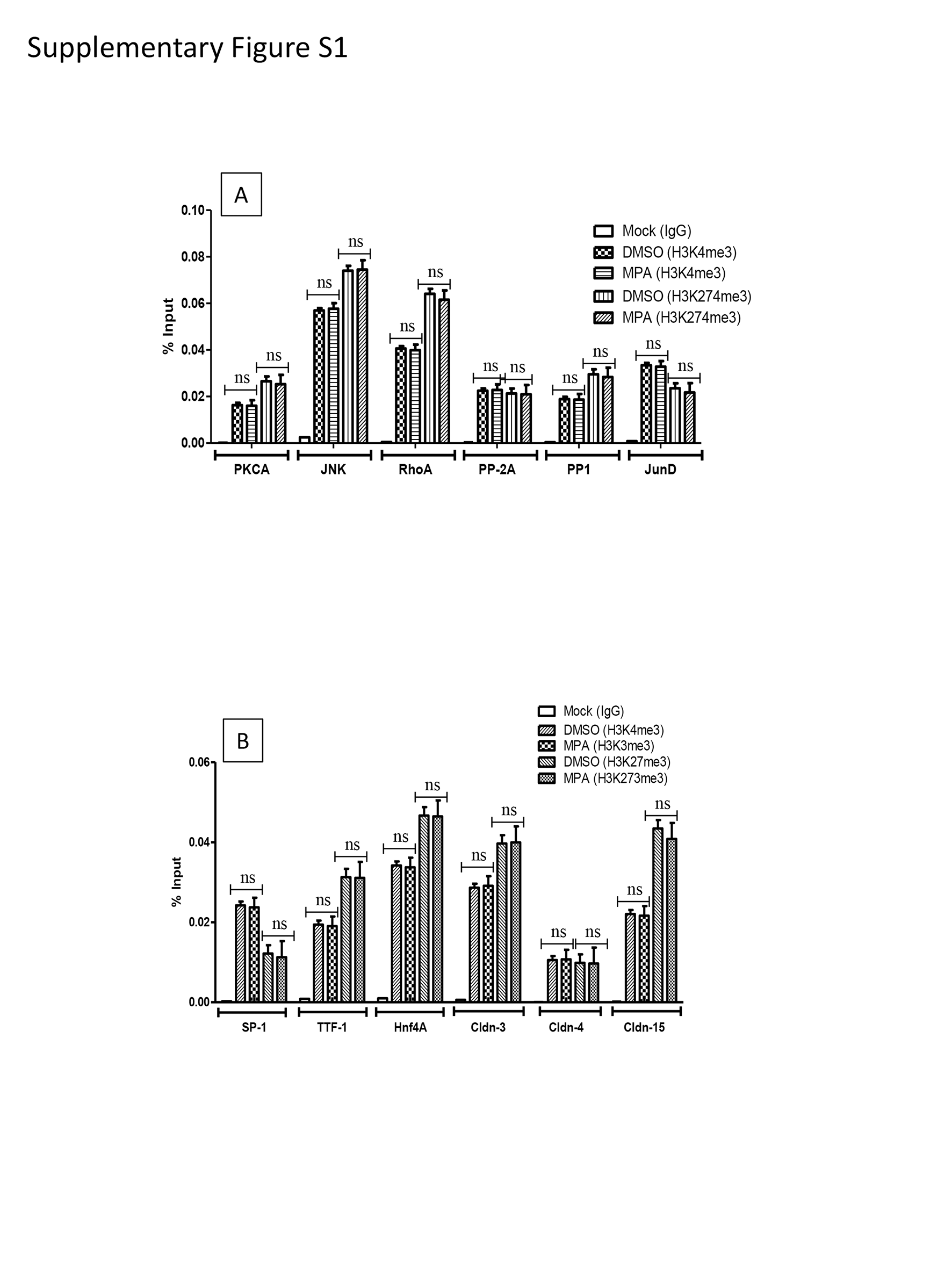

Supplement: Figure S1 — Regulatory effect of MPA treatment on the promoter of other genes involve in the regulation of TJ permeability. (A,B) Promoter activity of (PKCA, JNK, RhoA, PP-2A, PP1, JunD, Sp-1, TTF-1, Hnf4A, Cldn-3, -4, and Cldn-15) genes: ChIP assay was performed with antibodies specific to the activation mark (H3K4me3) or repression mark (H3K27me3) or IgG, followed by real time PCR analysis. Data was analyzed using % input to calculate the respective relative intensity of activation or repression mark in the MPA treated cells as compared to control (DMSO) cells. Differences between groups were analyzed by ANOVA with Bonferroni post-test. The values were expressed as means ± SEM (n = 3). Whereas, ns, non-significant. [file Image1.TIF]

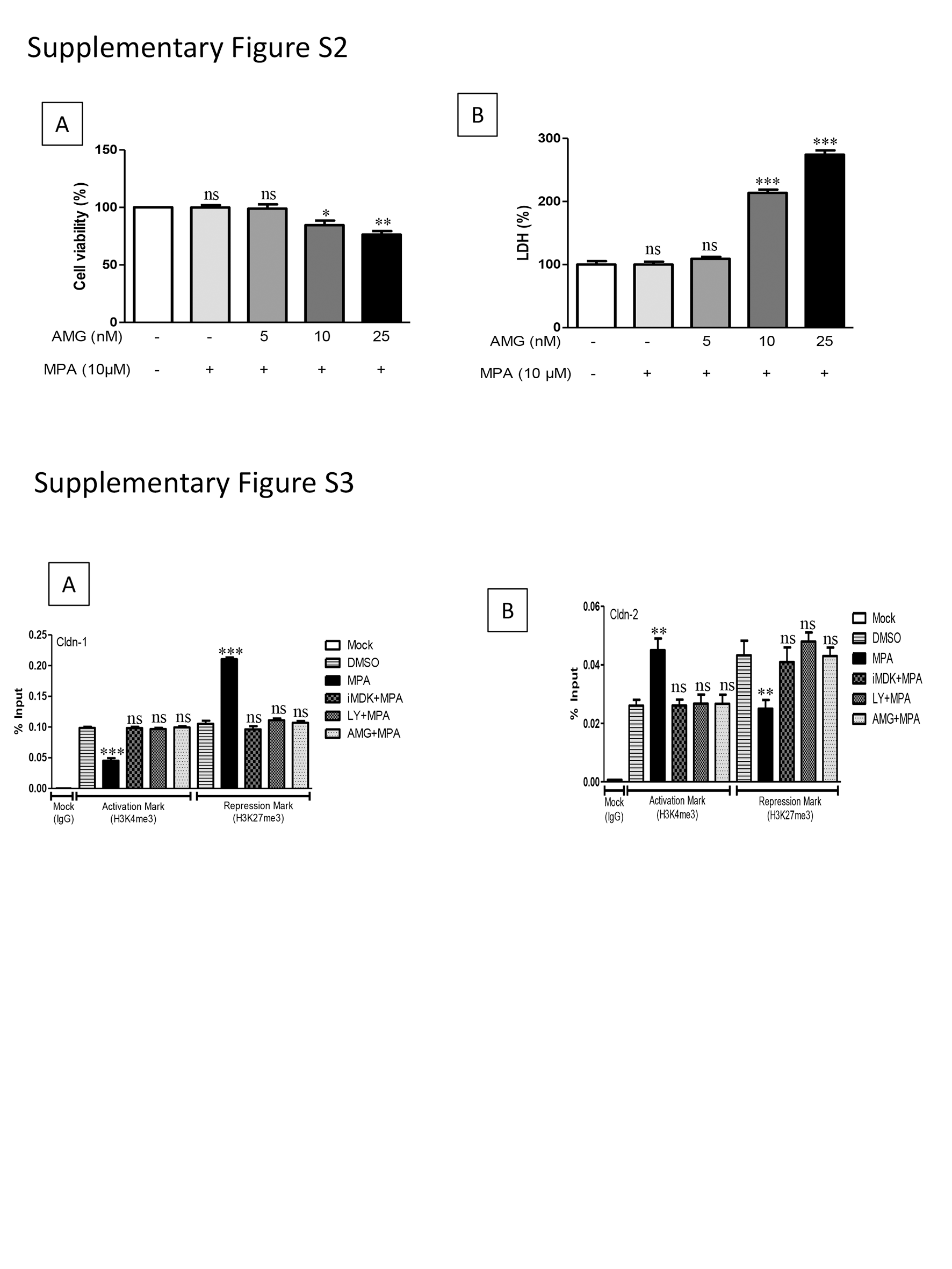

Supplement: Figure S2 — Cytotoxic effects of PI3K inhibitor (AMG) in co-treatment with MPA on Caco-2 cells. (A,B) Caco-2 cells were grown for 13 days post-confluence and treated with MPA or AMG + MPA or DMSO for 72 h. Cellular viability was measured using trypan blue exclusion assay after 72 h of treatment. Results are expressed as percentage of DMSO control (100%). (A) Cell viability was further confirmed by measuring the LDH release in the culture media in percentage to the DMSO control (100%). (B) Differences between groups were analyzed by ANOVA with Bonferroni post-test. The values were expressed as means ± SEM (n = 3). Whereas, *P < 0.05, **P < 0.01, ***P < 0.001, and ns, non-significant. [file Image2.TIF]
