# Supplementary material for: MPA Modulates Tight Junctions' Permeability via Midkine/PI3K Pathway in Caco-2 Cells: A Possible Mechanism of Leak-Flux Diarrhea in Organ Transplanted Patients
Source: Front Physiol. 2017 Jun 26;8:438. doi: 10.3389/fphys.2017.00438 (PMC5483464; doi:10.3389/fphys.2017.00438)
Supplement: Table S1 — List of primers used in this study. [file Table1.docx]

| **Types** | **Primer name** | **Chromosomal**  **location** | **Direction** | **Sequence** |
| --- | --- | --- | --- | --- |
| **Promoter Assay Primers** | PI3Kgamma | 7 | Forward | CGGCAATAGTTTTGCAGGTG |
|  |  |  | Reverse | GGTTTCCTTCATGGTTGAGG |
|  | CDX-2 | 13 | Forward | AAGGTTTACACTGCGGAAGC |
|  |  |  | Reverse | TCATACCACACCCTGTGCAT |
|  | Cldn-1 | 3 | Forward | GGACAGGATCTGACTCACCA |
|  |  |  | Reverse | CATCTCCTGGCATCCTCTTC |
|  | Cldn-2 | X | Forward | TCTCTTGGCCTCCAACTTGT |
|  |  |  | Reverse | CTGTGTGTGGCACATTCCAT |
|  | P38MAPK | 6 | Forward | TTTGACTCTTTCCCCGACAC |
|  |  |  | Reverse | AACTGGAGACCAAAGGCAGA |
|  | PKCA | 17 | Forward | GGACCATGGCTGACGTTTTC |
|  |  |  | Reverse | CGGCACCTACCAGATGAAGT |
|  | JNK | 10 | Forward | CTAATCAAGGCTCTGCGGTA |
|  |  |  | Reverse | ACCAAGAAGCTGCAAGATGC |
|  | RhoA | 3 | Forward | ATGGGTGGCACTCAGTCTCT |
|  |  |  | Reverse | ATGGGTGGCACTCAGTCTCT |
|  | PP-2A | 9 | Forward | AATCTCCTGCTCTGCCAAAC |
|  |  |  | Reverse | AGCGAAGATGTTAGCCTTCG |
|  | PP-1 | 10 | Forward | CCCTGAACAATTCCGTCACT |
|  |  |  | Reverse | CCCTGAACAATTCCGTCACT |
|  | JunD | 19 | Forward | TTTCTCTCCTCCCTCTGTCC |
|  |  |  | Reverse | GCAGATCAAAGACCCCAAGA |
|  | SP-1 | 12 | Forward | GCCGTTGTTCTGTCATTCCT |
|  |  |  | Reverse | TGGTGTCCGCCTAAAAAGAC |
|  | TTF-1 | 14 | Forward | TGGGAGGATCTTGTCTTTGG |
|  |  |  | Reverse | TGGTATTTCGGTCCTCCACT |
|  | Hnf4A | 20 | Forward | CCCAGAACAAGGATCCAGAA |
|  |  |  | Reverse | CCCCAAGTCAGGCATTCTAA |
|  | Cldn-3 | 7 | Forward | CCCAAAGTGGTGAGGAGAGA |
|  |  |  | Reverse | CCCAAAGTGGTGAGGAGAGA |
|  | Cldn-4 | 7 | Forward | TGACAAAAACCCCTCCCTCT |
|  |  |  | Reverse | ACGGACTTAACGTTCGCAGA |
|  | Cldn-15 | 7 | Forward | GTTCAAGCAATCGTCTCAGC |
|  |  |  | Reverse | CATCCATTCACCAGGGAACT |
|  | GAPDH | 12 | Forward | TGAGCAGTCCGGTGTCACTA |
|  |  |  | Reverse | ACGACTGAGATGGGGAATTG |
| **Expression Primers** | PI3Kgamma | 7 | Forward | CCAAGGAAGCTTCAATGCTGAC |
|  |  |  | Reverse | TCCTCTGCTGTGAGAGGGTTAA |
|  | CDX-2 | 13 | Forward | GAACCTGTGCGAGTGGATG |
|  |  |  | Reverse | AAGGGCTCTGGGACACTTCT |
|  | Cldn-1 | 3 | Forward | CAGTGGAGGATTTACTCCTATGC |
|  |  |  | Reverse | GTGGCAACTAAAATAGCCAGACC |
|  | Cldn-2 | X | Forward | CCAGCATTGTGACAGCAGTT |
|  |  |  | Reverse | TCATGCCCACCACAGAGATA |
|  | P38MAPK | 6 | Forward | CCAGCTTCAGCAGATTATGC |
|  |  |  | Reverse | TGGTACTGAGCAAAGTAGGCA |
|  | GAPDH | 12 | Forward | ACCCAGAAGACTGTGGATGG |
|  |  |  | Reverse | TTCTAGACGGCAGGTCAGGT |

**Supplementary Table 1: List of primers used in this study**
